# Evaluation of Inner Exposure of Horses to Zearalenone (ZEN), Deoxynivalenol (DON) and Their Metabolites in Relation to Colic and Health-Related Clinical-Chemical Traits

**DOI:** 10.3390/toxins13080588

**Published:** 2021-08-23

**Authors:** Sven Dänicke, Janine Saltzmann, Wendy Liermann, Maren Glatter, Liane Hüther, Susanne Kersten, Annette Zeyner, Karsten Feige, Tobias Warnken

**Affiliations:** 1 Institute of Animal Nutrition, Friedrich-Loeffler-Institut, Federal Research Institute for Animal Health, Bundesallee 37, 38116 Braunschweig, Germany; janine.saltzmann@fli.de (J.S.); liane.huether@fli.de (L.H.); susanne.kersten@fli.de (S.K.); 2 Institute of Nutritional Physiology “Oskar Kellner”, Leibniz Institute for Farm Animal Biology, Wilhelm-Stahl-Allee 2, 18196 Dummerstorf, Germany; liermann@fbn-dummerstorf.de; 3 Group Animal Nutrition, Institute of Agricultural and Nutritional Sciences, Martin Luther University Halle-Wittenberg, Theodor-Lieser-Str.11, D-06120 Halle/Saale, Germany; maren.glatter@landw.uni-halle.de (M.G.); annette.zeyner@landw.uni-halle.de (A.Z.); 4 Clinic for Horses, University of Veterinary Medicine Hannover, Bünteweg 9, 30559 Hannover, Germany; karsten.feige@tiho-hannover.de (K.F.); tobias.warnken@tiho-hannover.de (T.W.)

**Keywords:** zearalenone, deoxynivalenol, metabolism, horse, health, intestinal disorders, colic

## Abstract

Mycotoxin contaminated feed has been associated with colic of horses caused by intestinal disorders. Whether such disease conditions alter the intestinal toxin metabolism and transfer across a compromised mucosal barrier is unknown. A screening approach was used to relate blood residue levels of DON, ZEN and their metabolites to the status of the horses (sick vs. healthy). A total of 55 clinically healthy horses from 6 different farms with varying feeding background served as control for sick horses (N = 102) hospitalized due to colic. ZEN, alpha-zearalenol (ZEL), beta-ZEL and DON were detectable in peripheral blood as indicators for the inner exposure with significant farm effects for alpha- and beta-ZEL. However, the levels in sick horses were similar to all farms. Moreover, the proportion of beta-ZEL of all detected ZEN metabolites as an indicator for the degree of metabolism of ZEN was not different for sick horses but differed amongst the control farms. Although the incidence of DON in blood was generally low and not significantly different amongst healthy and sick horses, the positive samples were nearly exclusively found in sick horses suggesting either a higher toxin transfer, an association of DON with the development of colic or a different feeding background.

## 1. Introduction

As herbivores, horses are continuously exposed to various mycotoxins formed by several fungal species, most notably by *Fusarium spp*., which infect many plants used as feedstuffs. Deoxynivalenol (**DON**) and zearalenone (**ZEN**) are of outstanding importance due to their frequent occurrence in plant originating feedstuffs including horse feed [1,2]. Here, parts of the plants, such as grains and straw, may vary in mycotoxin distribution [3,4,5] or feedstuffs change toxin profile in the course of preservation [6] and storage [7]. Therefore, depending on the ration type, i.e., the proportion of concentrate feed, the exposure of horses to DON and ZEN is expected to vary accordingly. In addition, straw used as bedding material or as feedstuff might pose an additional source of inhalative and oral exposure to these and other toxins [8,9]. Besides the frequency of occurrence and levels of DON and ZEN in the environment of horses their toxicological relevance is further determined by the sensitivity of this species to these toxins. As observed particularly in pigs [10], DON is also supposed to adversely affect feed intake by horses, although experimental findings are inconsistent [11,12,13,14,15]. Besides feed intake, liver lesions and general health problems including colic are supposed to be associated with DON exposure of horses [16,17]. Commonly, the term colic summarizes abdominal pain of horses [18] irrespective of etiopathology but with acute gastrointestinal diseases as the most common causes [19].

Gastrointestinal originating colic is often linked, amongst others, with disturbed motility and perfusion of the intestines, an associated compromised mucosal barrier giving rise to a forced transfer of chyme originating endotoxins [20,21,22], and possibly mycotoxins, across the damaged mucosa. If it is further considered that mycotoxins and particularly DON have been implicated in intestinal inflammation and in affecting intestinal epithelium turnover, metabolism and protein synthesis, including that of tight junction proteins [23,24], it is conceivable that mycotoxin actions could be a factor contributing to the development of colic. Thus, mycotoxins could not only be a factor contributing to the development of colic through inducing intestinal lesions and inflammation but also support their own transfer though a damaged epithelium at the same time.

Besides a proposed increased mycotoxin transfer, the inner exposure to the toxins might be further modified under such conditions through influencing their metabolism generally mediated by gastrointestinal microbiota and mucosa, as well as by the liver [25,26]. Gastrointestinal originating colic is known to alter the chyme microbiome [18], which might have consequences for its capacity to metabolize DON to the less toxic de-epoxy-DON, and ZEN to beta-zearalenol (beta-ZEL) and further metabolites as commonly detected metabolites in horses [11,27,28]. Thus, gastrointestinal disorders might modify the toxicity of DON and ZEN compared to healthy horses through an altered metabolism even at similar dietary exposure.

A conceivably ensuing endotoxemia might trigger a systemic inflammatory response resulting in an increased ratio between kynurenine (**Kyn**) and tryptophan (**Trp**) in peripheral blood. Kyn is the main degradation product of the amino acid Trp. This reaction is catalyzed by indolamine 2,3-dioxygenase (**IDO**), an ubiquitous occurring enzyme, which is mainly induced by pro-inflammatory stimuli [29,30] released upon endotoxin contact with competent immune cells. The same reaction is also catalyzed by the liver-specific tryptophan 2,3-dioxygenase (**TDO**) [31]. As the conversion of Trp to Kyn is catalyzed by IDO/TDO during only a single step, the ratio between Kyn and Trp in systemic blood is frequently regarded as an indicator for those enzyme activities. An induced IDO-mRNA and an increased Kyn to Trp ratio have been described for pigs during a systemic inflammatory response induced by infusion of lipopolysaccharides (**LPS**) as endotoxin source [32]. In horses, Kyn and the Kyn to Trp ratio have also been linked to inflammatory conditions induced by an oral glucose tolerance test [33,34]. Thus, it might be hypothesized that intestinal disorders are related to an increased Kyn to Trp ratio. However, as Trp is not only degraded to Kyn but primarily used to meet the Trp requirement for protein synthesis [35] the interpretation of the ratio might be confounded by fluctuations in Trp content in blood driven by absorption from the intestine and usage in pathways others than degradation to Kyn; e.g., protein synthesis.

The liver as a central metabolic and secondary immunological organ is implicated in metabolism of DON and ZEN [25,26] In addition, this organ is involved in clearance of endotoxins increasingly drained from a disordered gut, and in mediating the inflammatory response and is thus target itself not only from the mycotoxin action but also of the inflammation. Hence, liver lesion-indicating enzyme activities in peripheral blood could be associated to mycotoxin exposure and intestinal disorders.

Based on the hypothesized associations between intestinal disorders, inflammation and liver lesions possibly triggered by a forced endotoxin and mycotoxin transfer across the intestinal barrier we aimed at examining the inner exposure to DON, ZEN and their metabolites of horse patients hospitalized due to colic with an unknown feeding anamnesis and to compare this exposure with that of clinically healthy counterparts from 6 different farms (**Screening**). As blood profiles of DON, ZEN and their metabolites vary with feed contamination background, various farms were sampled to cover a range of different feeding practices. By relating blood levels of DON, ZEN and their metabolites of horse patients to liver lesion and inflammation indicators with those obtained from clinically healthy horses, we aimed to identify the relevance of these mycotoxins for intestine disorders under practical feeding conditions.

Another aim was to scrutinize the time-dependent progression of these toxins under controlled feeding conditions to obtain information on the relationships between intake of contaminated feed and blood levels of toxin residues at various time points relative to intake in order to evaluate the reliability of collecting only spot blood samples without information on the last feed intake (common diagnostic practice) (**Kinetics**).

Next, we aimed to get further insight into which factors could influence the Kyn to Trp ratio in horses, other than inflammation, for a better understanding of the ratios determined for horses with intestine disorders. For this, blood samples were analyzed, which were frequently collected from healthy horses at defined time points after a (Trp containing) morning meal (**Kinetics**).

## 2. Results

### 2.1. Screening

#### 2.1.1. Mycotoxin Residues

Besides the parent compounds ZEN and DON, their metabolites alpha-zearalenol (**alpha-ZEL**), beta-zearalenol (**beta-ZEL**), zearalanone (**ZAN**), alpha-zearalanol (**alpha-ZAL**), beta-zearalanol (**beta-ZAL**) and de-epoxy-deoxynivalenol (**DOM-1**) were included in the multi-method. However, only ZEN, alpha- and beta-ZEL and DON were detectable in the samples.

**Figure 1:**
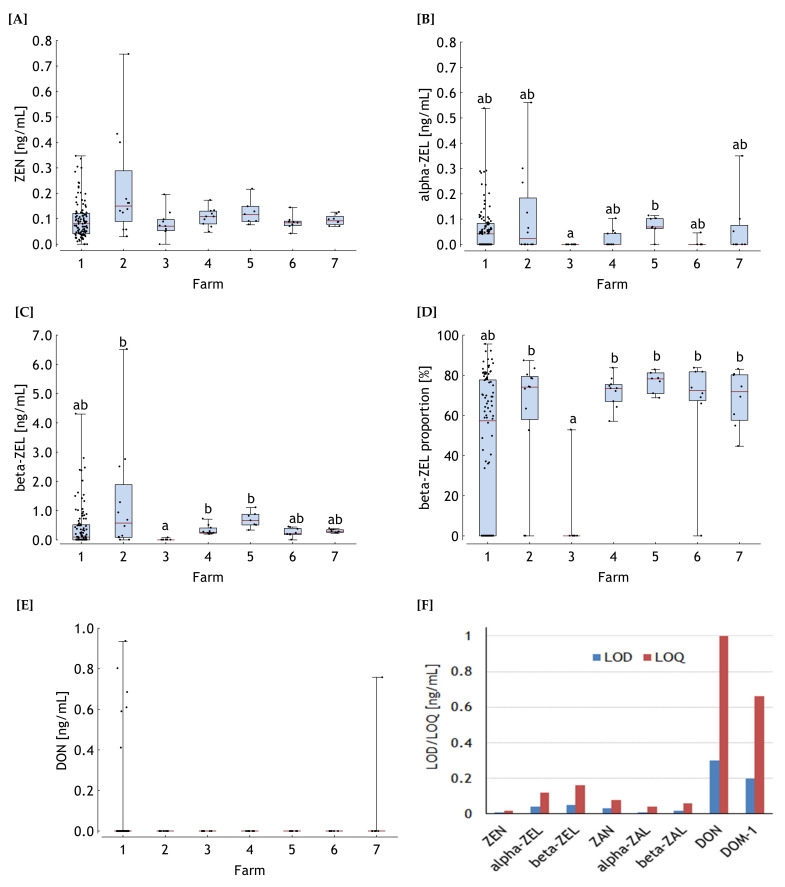
Zearalenone (ZEN) (**A**), alpha-zearalenol (alpha-ZEL) (**B**) and beta-ZEL (**C**) concentrations in blood serum of horses, proportion of beta-ZEL of the sum of ZEN, alpha- and beta-ZEL (**D**) and serum deoxynivalenol (DON) concentrations (**E**) in horses hospitalized for colic (Farm 1, *n* = 102) and of clinically healthy horses from 6 different farms (Farm 2 to 7, *n* = 7–12). Boxes represent the span between the 25th and 75th percentile, the horizontal line within the boxes the median and the whiskers the range between minimum and maximum values. ab, distributions not sharing similar superscripts differ significantly (*p* < 0.05). Limits of detection (LOD) and of quantification (LOQ) are shown in (**F**) for ZEN, alpha-ZEL, beta-ZEL, zearalanone (ZAN), alpha-zearalanol (alpha-ZAL), beta-ZAL, DON and de-epoxy-DON (DOM-1).

ZEN was detected in all samples from farms 2 and 4 to 7, while 97% and 90% of the analyzed samples from farm 1 and 3 were detected positively for ZEN. The statistical evaluation revealed no significant differences amongst farms according to the Kruskal–Wallis test (*p* > 0.05) (Figure 1A). The median of all samples amounted to 0.09 ng/mL and the maximum concentration of 0.75 ng ZEN/mL was detected in farm 2.

In contrast, the concentrations of alpha-ZEL were significantly different between farms (*p* < 0.05) (Figure 1B). Alpha-ZEL remained undetectable in samples collected from farm 3 (0% positive samples) while the proportions of positive samples varied markedly amongst the other farms and amounted to 52%, 50%, 40%, 86%, 13% and 38% in farms 1, 2, 4, 5, 6 and 7, respectively. Post-hoc evaluation demonstrated that samples from farms 3 and 5 differed significantly while samples from all other farms differed neither to each other nor to farm 3 and 5. In principle, similar significance relationships were observed for beta-ZEL with the exception that not only samples from farm 5 differed from farm 3 but also from farms 2 and 4 (Figure 1C). The proportions of samples positively detected for beta-ZEL were 58%, 83%, 10%, 100%, 100%, 88% and 100% for farms 1 to 7, respectively.

The proportions of beta-ZEL of the sum of ZEN, alpha- and beta-ZEL were lower in farm 3 and varied less compared to the other farms as indicated by a median of 0% (Figure 1D). Thus, this proportion from farm 3 differed significantly from farms 2, 4, 5, 6 and 7 while farm 1 was characterized by the largest variation and reached a maximum proportion of 96% and was not significantly different from any other farm.

Seven out of the 157 samples were detected positively for DON with farms 2 to 6 completely being negative (Figure 1E). Six samples with DON concentrations ranging between 0.4 and 0.9 ng/mL were detected for farm 1 (6% positive) while a single DON concentration of 0.8 ng/mL was found in serum of horses from farm 7 (13% positive). No significant farm effects were detected.

#### 2.1.2. Clinical-Chemical Traits

In general, the largest variation for all clinical-chemical indicators was found for the ill horses (farm 1).

**Figure 2:**
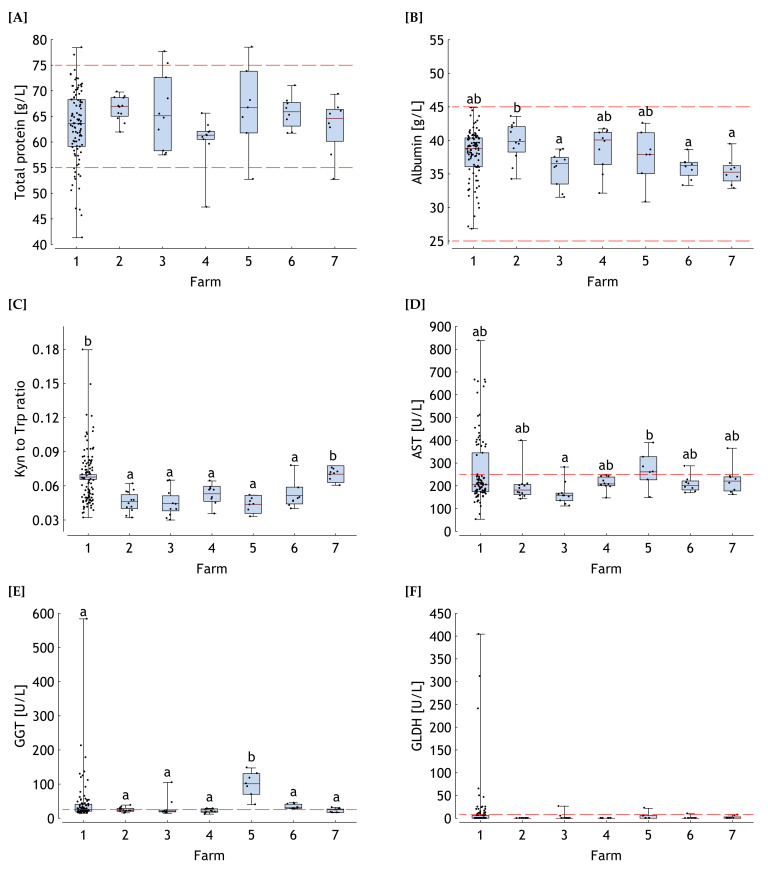
Total protein and albumin concentrations (**A**,**B**), kynurenine (Kyn) to tryptophan (Trp) ratio (**C**), aspartate amino transferase (**D**), gamma-glutamyl transferase (**E**) and glutamate dehydrogenase (**F**) activities in blood of horses hospitalized for colic (Farm 1, *n* = 102) and of clinically healthy horses from 6 different farms (Farm 2 to 7, *n* = 7–12). Boxes represent the span between the 25th and 75th percentile, the horizontal line within the boxes the median and the whiskers the range between minimum and maximum values except for the Kyn to Trp ratio, where the boxes represent the ± range of the standard deviation and the horizontal line the mean value. ab distributions or mean values not sharing similar superscripts differ significantly (*p* < 0.05). Reference ranges (minimum-maximum) or maximum reference values [36,37] are indicated by horizontal red broken lines.

Total protein varied from 41 to 78 g/L in sera of farm 1 with 14 samples dropped below the lower reference value of 55 g/L and 2 exceeding the upper bound of 75 g/L (Figure 2A). Samples from all other farms (healthy horses) only occasionally felt out of the reference range for total protein. Total protein content was not significantly influenced by farm.

Albumin concentration also varied largely in samples collected from farm 1 ranging between 27 and 45 g/L although these extreme values still were in the reference range (25–45 g/L) (Figure 2B). Similarly, samples of all other farms complied with this reference range. However, the albumin content was significantly lower in samples from farms 3, 6 and 7 when compared to farm 2 whilst the albumin levels from the other farms showed no significant differences to any of the farms.

**Figure 3:**
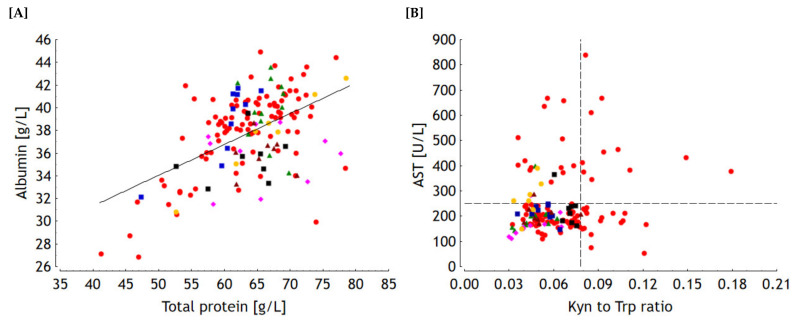
Serum albumin concentration in dependence on total protein content in serum (y = 20.5 + 0.27·x, r² = 0.27, *p* < 0.001, residual standard deviation = 3.0 g/L, N = 153) (**A**). Associations between aspartate amino transferase (AST) activity and kynurenine (Kyn) to tryptophan (Trp) ratio in serum in relation to the reference value for AST and to the highest Kyn to Trp ratio observed in healthy horses (broken lines) (**B**). Farm 1 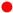, 2 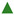, 3 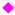, 4 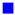, 5 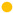, 6 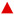, 7 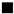.

The differences between these farms became also obvious when the albumin concentration was linearly regressed on the total protein content across all farms (Figure 3A). Here, the corresponding data-pairs of farm 2 fell mostly above the regression line while those of farms 3, 6 and 7 were predominantly below this line. This makes clear that farm-specific effects might contribute to the residual standard deviation of 3 g/L corresponding to a variance proportion of 73% not explainable by the linear regression. Irrespective of this variation, the slope of the linear regression line suggests an overall mean albumin content of 27% of total serum protein in horses.

The Kyn to Trp ratio was significantly higher in farms 1 and 7 and reached a maximum value of 0.18 in farm 1 (Figure 2C). Taking the maximum ratio of 0.078 from all healthy horses, i.e., from farms 2 to 7, as a reference point a total of 27% of all samples from farm 1 exceeded this ratio.

Farm effects were detected for aspartate aminotransferase (AST) activity in serum with farm 3 showing a significantly lower level compared to farm 5 while all other farms displayed comparable levels (Figure 2D). However, particularly in disordered horses from farm 1, a closer look to variation seems to be necessary for an adequate evaluation of individual cases. It is interesting to note that AST appeared to be clustered in ill horses with 25% exceeding the reference value of 250 U/L markedly and showing extreme values in the range between approximately 350 and 840 U/L. Although the correlation between AST and the Kyn to Trp ratio was proven to be significant (r = 0.18; *p* < 0.05), the associations appeared rather loose and seemed to separate ill from heathy horses (Figure 3B). Taking the cross between the horizontal line, representing the reference value of AST, and the vertical line indicating the highest Kyn to Trp ratio as a reference point, the horses would fall into four quadrantes representing different illness conditions. The 11 horse patients in the upper right quadrant were characterized by both higher AST and Kyn to Trp ratios representing supposedly a higher degree of muscular lesions due to more pronounced colic and inflammation at the same time. Another 16 horse patients imposed by inflammation (higher Kyn to Trp ratios), but less pronounced colic associated muscular activity were located in the lower right quadrant. Thirteen horse patients and some healthy horses showed increased AST activity without increased Kyn to Trp ratios. Finally, the remaining horse patients and healthy horses found in the lower left quadrant were inconspicuous with regard to AST activity and Kyn to Trp ratios.

Similar to AST, farm 5 imposed by the significantly highest gamma-glutamyl transferase (**GGT**) activity in serum compared to all other farms (Figure 2E). It is worth to note that all samples of this farm exceeded the reference value of 25 U/L. Approximately 50% of all samples collected from ill horses (farm 1) did not comply with this reference value either.

Although no significant differences in glutamate dehydrogenase (GLDH) activity were detected between farms some exceptionally high values in farm 1 need to be stressed with 22% of samples exceeding the reference value of 8 U/L (Figure 2F). Furthermore, it needs to be considered that in farms 2 and 4 all GLDH concentrations were lower than the test limit while 28%, 20%, 43%, 13% and 25% were detected positive in farms 1, 3, 5, 6 and 7, respectively.

#### 2.1.3. Principal Component Analysis (PCA)

For a further evaluation of the associations between the variables a correlation based PCA was performed. The first two components (PC1 and PC2) out of the 13 newly generated PC (variables) extracted approximately 37% of the total variance. The scree-plot of the eigenvalues did not indicate a distinct break point which would separate the more informative from the less important components. In such instances, the mean value of all eigenvalues of 1.0 usually serves as a separation criterion. Here, this mean value corresponded to a total of 6 extracted components which explained approximately 78% of the total variance.

**Figure 4:**
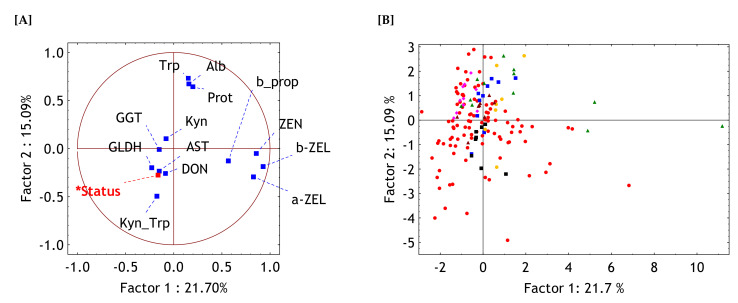
Visualization of the results of the principal component analysis. Shown are the projection of the variables and of the status (healthy or intestine disorders) (**A**), and of the cases (individual horses) (**B**), respectively, into the space spanned between the first two components (PC or factors). Abbreviations for serum variables: GGT = gamma-glutamyl-transferase; GLDH = glutamate dehydrogenase; AST = aspartate aminotransferase; Prot = total protein; Alb = albumin; Trp = tryptophan; Kyn = Kynurenine; Kyn_Trp = ratio between Kyn and Trp; a-ZEL = alpha-zearalenol (ZEL); b-ZEL = beta-ZEL; ZEN = zearalenone; b_prop = proportion of b-ZEL of the sum of ZEN, a- and b-ZEL; DON = deoxynivalenol. Farm 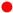, 2 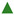, 3 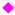, 4 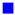, 5 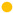, 6 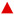, 7 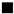.

To visualize the relationships between all 13 variables they were plotted in the space spanned between the most informative new variables PC1 and PC2 (Figure 4A). As the PCA was based on correlations a localization of a specific variable in the center formed by the cross between the zero-correlations of PC1 and PC2 means a poor or no correlation of that variable to both components. On the other hand, localizing at the circle indicates a correlation to either PC1 or PC2 of 1.0 or −1.0.

Trp, total protein and albumin concentrations formed a cluster and were moderately positively correlated to PC2 while ZEN and its derivatives formed another cluster but correlated positively to PC1. The third cluster of variables comprised of GLDH, GGT, AST, Kyn, Kyn to Trp ratio and DON and was closely associated to the status of the horses which included sick horses (farm 1) and healthy horses (farms 2–7).

Plotting the cases, i.e., the individual horses, in the same variable space between PC1 and PC2 suggested cluster forming particularly for the horses from the control farms which were predominantly situated in the upper two quadrantes (Figure 4B). In contrast, most sick horses were in the lower two quadrantes although some of these horses were located in the upper left quadrant. MANOVA revealed a significant farm effect (*p* < 0.001) and a significant effect of the status of the horses (all healthy horses from farms 2 to 7 vs. sick horses) (*p* < 0.001). As the latter grouping comprised only two expressions, it can be concluded that the cluster formed by sick horses differed significantly from that produced by the healthy ones.

### 2.2. Kinetics

#### 2.2.1. Mycotoxins

**Figure 5:**
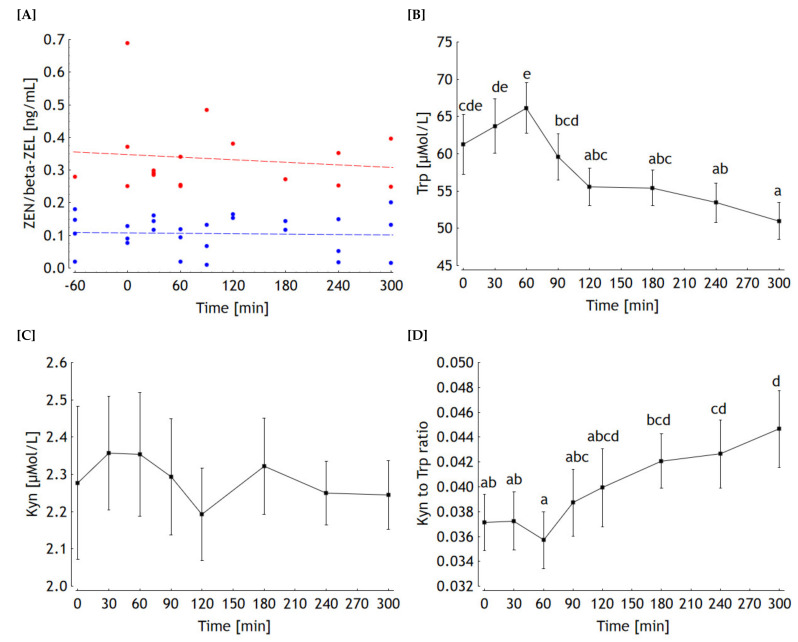
Time profiles of zearalenone (ZEN, in blue) and beta-zearalenol (beta-ZEL, in red) concentrations in blood of horses fed a ration containing 19 µg ZEN and 512 µg DON/kg feed corresponding to an exposure of 53 (±4) ng ZEN/kg body weight (bw) and 1420 (±3) ng DON/kg bw, respectively (**A**). Exposure was calculated based on this single morning meal given at −60 min as hay, and at 0 min as concentrate feed. Blood was sampled before offering these feed components and frequently until 300 min after offering the concentrate feed proportion (*n* = 5). Only values higher than the limits of quantification (LOQ) were plotted. Slopes of the linear regressions were not significantly different from zero (*p* > 0.05). Tryptophan (Trp) (**B**) and kynurenine (Kyn) (**C**) concentrations and the resulting molar ratio of Kyn to Trp (**D**) in blood of the same horses. Squares represent the lsmeans and whiskers the standard errors of the lsmeans (*n* = 5); a–e lsmeans not sharing similar superscripts differ significantly (*p* < 0.05).

DON was detectable in only 2 samples (1.27 and 0.53 ng/mL at −60 and 240 min, respectively, different horses). Alpha-ZEL was detected in only 1 individual sample with a concentration of 0.07 ng/mL at −60 min. The proportions of positive samples were higher for ZEN and beta-ZEL and amounted to 48% and 31%, respectively and were based on different horses and time points. The corresponding individual concentrations were plotted against time relative to offering the concentrate feed (Figure 5A). Neither for ZEN nor for beta-ZEL a time dependency could be detected as indicated by the slopes of the corresponding linear regression lines not being significantly different from zero (Figure 5A).

#### 2.2.2. Trp, Kyn and Kyn to Trp Ratio

The Trp and Kyn concentrations at −60 min were different amongst horses and thus served as a co-variate for the ANOVA and the following post-hoc test. Trp concentration increased significantly until 60 min after offering the concentrate feed at time 0 (Figure 5B). Thereafter, a steep decrease until 120 min was followed by a decelerated decrease for the remaining observation period until 300 min. In contrast, the Kyn concentration remained stable over time and varied inconsistently (Figure 5C). Based on the kinetics of Trp and Kyn the resulting progression of the Kyn to Trp ratio was driven by the Trp time-dependent concentration profile. After a lag phase until 30 min the ratio decreased to its lowest mean concentration at 60 min and increased continuously thereafter. From time 180 min onwards, the ratio was significantly higher compared to its nadir (Figure 5D).

## 3. Discussion

The aim of the present screening was to test the hypothesis that DON and ZEN present in horse feed at background levels would be increasingly transferred across intestinal epithelia putatively damaged through intestinal disorders resulting in an enhanced inner exposure to these toxins and their metabolites. In addition, as particularly large intestinal disorders might affect intestinal microbes which are a major source of mycotoxin metabolism an altered DON and ZEN metabolite profile contributing to the inner exposure is conceivable. Such a screening approach relies on a larger pool of samples covering a broad spectrum of feeding conditions and thus enable comparison between sick and healthy horses. The significant farm effects on alpha-ZEL, beta-ZEL and the proportion of beta-ZEL of all ZEN metabolites in blood of healthy horses principally confirms our assumption that different feeding background is reflected in mycotoxin blood profiles. However, a further discussion of the blood levels in the context of used feedstuffs (Table 1) is hampered by a lack of information of intake amounts; a typical situation in practical horse feeding, particularly if horses have access to pasture. It can at least be stated that pasture as the sole feed source does not necessarily results in the lowest blood mycotoxin levels.

Horses of farm 3 showed the lowest levels of alpha- and beta-ZEL with a similar trend for ZEN when compared to some of the control farms. However, the corresponding concentrations measured in sick horses were similar to all control farms due to the high individual variation at comparable medians. The apparently higher variation in sick horses might be caused by the fact that all of these 102 horses were assigned to farm 1 (clinic for horses) although they originated from different farms with putatively varying feeding background. Moreover, in case of DON and ZEN which are characterized by a rapid elimination from the body the time of last feed intake needs to be considered in interpreting of diagnostic results. All the horse patients were hospitalized due to clinical symptoms of colic and blood samples were collected upon arrival in the clinic without information on last feed intake. However, time at blood sampling relative to the last feed intake does obviously not play a major role in the measured blood levels since horses from the kinetic study showed stable levels of ZEN and beta-ZEL at least until 5 h after offering feed. This conclusion is further supported by the longer period of time to reach maximum blood concentrations of ZEN and its metabolites after a ZEN containing meal of 8 to 12 h which is considerably longer compared to pigs [26,28]. Moreover, under steady state feeding conditions and offering the ZEN-contaminated oats two times per day relatively stable blood levels were detected [28]. This delayed ZEN-kinetics might be due to feeding practice adjusted to the feeding behavior of the horse, i.e., offering hay or other roughage for long periods of the day. Whether this primarily roughage-based feeding shifts the absorption and microbial mycotoxin metabolism more to the hindgut cannot be answered currently. Besides intestinal microbiota, the intestinal mucosa and the liver are involved in ZEN metabolism [26] while DON was shown to be metabolized to DOM-1 nearly exclusively by intestinal microbes [25]. The fact that only DON was detected in blood although the LOD and the LOQ of DOM-1 were lower than those for DON would rather favor the small intestine as absorption site for non-metabolized DON. This conclusion is further supported by a dose-response experiment testing dietary DON-levels up to approximately 7.3 mg/kg [11]. Under these conditions, the DOM-1 concentration in blood paralleled the dose-dependent increase in DON content but at an approximately 4-fold lower level.

Based on the linear relationships between DON-exposure and DON in blood as reported by Schulz et al. [11] the estimated DON-concentrations in feed of the horses of the present study would be expected at background contamination between ~0.3 and 0.5 mg DON/kg feed based on the detected blood levels between 0.4 and 0.9 ng/mL.

Amongst the healthy horses (farm 2 to 7) only one blood sample was detected positively for DON (1.8% positive rate) compared to a 6% positive rate in the sick horses. This difference between healthy and sick horses needs to be discussed with caution in the view of the general low DON incidence in blood. Nevertheless, the higher proportion of positive DON samples in sick horses could confirm the initial hypothesis that colic associated intestinal mucosal lesions could facilitate the transfer of DON from chyme to peripheral blood. On the other hand, the higher DON incidence could simply reflect a feeding background different from that of the healthy horses. This raises the question whether this other feeding background could have been a factor contributing to the development of a colic. Poor hygienic feed, including contamination with DON and other mycotoxins, has been associated with colic of horses [8,16,17,38,39].

The uncertainties in discussing the mycotoxin residues in blood without additional information on feed mycotoxin contents might partially be counterbalanced by interpreting blood metabolite contents relative to the sum of the parent compound and all detected metabolites as an indicator for the efficiency of overall metabolism, including microbial, intestine mucosal and hepatic metabolism. As all serum samples were treated with β-glucuronidase prior to analysis such a discussion includes both free and glucuronidated parent toxins and derived metabolites. In the present screening such a discussion is only possible for ZEN as the DON metabolite DOM-1 remained undetectable as outlined above. The absence of DOM-1 despite a lower LOQ compared to DON supports the view that DON is absorbed in its non-metabolized form in the small intestine leaving only small amounts of DON to be metabolized by colonic microbiota. Indeed, the proportion of DOM-1 in horse blood accounts for only 20% to 25% of that of DON after prolonged feeding a DON-contaminated diet [11].

Sick horses showed the largest variation in ZEN metabolization to beta-ZEL and covered the range between 0% and 96% which might be discussed as a broad spectrum of colic influences on intestinal microbiota as an important site in overall metabolism of ZEN in horses. Whether the cases with a particularly low or high ZEN metabolization degree indicate colic conditions with significantly altered intestinal microbiota cannot be answered by the current screening approach.

Colic initiates an acute phase reaction (**APR**) in horses with ceruloplasmin (**Cp**) and serum amyloid A (**SAA**) being prominent acute phase proteins (**APP**) [40]. Albumin which is known as a negative reactant of APR in many species [41] remained uninfluenced by colic. Blood albumin is a major constituent of total protein content and accounts for approximately 27% for the present data pool irrespective of the health status (Figure 3A). Although there were no significant differences for albumin and total protein between sick and healthy horses the larger span of individual values needs to be stressed. From a clinical viewpoint, hyperprotemia is sometimes interpreted as an indicator for hemoconcentration which can, however, be masked by a protein and albumin loss into the peritoneal cavity ultimately leading to a hypoproteinemia [20].

Endotoxemia is a common feature of horses presenting with colic and is paralleled by a stimulation of hepatic Kupffer cells and circulating monocytes giving rise to the release of inflammatory mediators triggering APR and also hepatocellular injury with the subsequent release of GLDH, GGT and AST [20,21,22]. However, under colic conditions, increased blood activities of AST are often paralleled by increases in creatinine kinase (CK) suggesting the involvement of musculature resulting from muscular traumata associated with colic induced behavior of affected horses, first aid intramuscular injections and transportation to the clinic [42]. Indeed, increased CK and AST activities have been linked to intestinal ischemia [42] which in turn might compromise intestinal barrier and consequently facilitate the transfer of DON and endotoxins giving rise to systemic inflammation. The Kyn to Trp ratio responds not only to inflammation [29,30,33,34,43] but also to feed and consequently Trp intake [44,45] as can be clearly deduced from the kinetic study. Here, the ratio progressed inversely to that of Trp with no changes in Kyn at the same time. Thus, the time-dependent changes in Trp content reflected rather metabolic pathways others than IDO/TDO mediated Trp degradation such as protein synthesis. Moreover, the distribution of the Kyn to Trp ratios within the healthy horses of the screening (farms 2–7) and of the kinetic study was comparable (mean: 0.04 vs. 0.04, minimum: 0.03 vs. 0.03, maximum: 0.056 vs. 0.078) suggesting that values higher than the overall maximum ratio of 0.78 observed in the pool of healthy horses could indeed indicate inflammatory conditions. Thus, in the present screening, a total of 27 horses were characterized by an inflammatory condition (see Figure 3B) when the threshold of the Kyn to Trp ratio of 0.078 is considered as a separating hallmark. Indeed, the two cases diagnosed with colitis felt in this group while the other 25 horse patients might have developed a systemic inflammation as a result of gastro-intestinal disorder related disturbances in gut motility, mucosal barrier and intestinal microbial communities with consecutive endotoxin transfer and triggering of an APR as discussed above. Higher AST activities without inflammation, i.e., Kyn to Trp ratios lower than 0.078 (left upper quadrant in Figure 3B), might indicate milder types of gastro-intestinal disorders or another stage of disease. It should be stressed that higher AST activities are also seen in cases of involvement of the liver. However, in the view that AST correlated only weakly with the more liver-specific GLDH and GGT it seems reasonably to discuss the AST variation in the context with colic associated muscular lesions.

## 4. Conclusions

Interpreting all recorded parameters collectively by using PCA it can be clearly deduced that AST, GGT, GLDH, DON, Kyn and Kyn to Trp ratio were closely associated to the status of the horses and separated ill from healthy horses. In contrast to DON, ZEN and its metabolites were less related to the status of horses which might be interpreted as a hint at different features of DON and ZEN in relation either to the outcome or development of colic.

## 5. Materials and Methods

### 5.1. Screening

#### 5.1.1. Horse Patients with Colic

Blood samples were leftovers from samples collected on a routine basis when horses were hospitalized in the Clinic for Horses, University of Veterinary Medicine Hannover, due to acute colic. Diagnoses were acute colic large (*n* = 13) and small (*n* = 3) intestine impaction, bloated colon and cecum (*n* = 11), small intestine dilatation (*n* = 3), colon displacement (*n* = 35), *torsio coli* (*n* = 1), gastric overfill (*n* = 1), *hernia mesenterialis* (*n* = 2), colitis (*n* = 2) and colic due to other causes (*n* = 31).

A feeding anamnesis was not available. Time between first signs of colic and blood sample upon arrival in the clinic was not known. For reasons of statistical evaluation these horses were assigned to farm 1 (Table 1).

#### 5.1.2. Clinically Healthy Horses

For screening of exposure, leftovers from blood samples collected for diagnostic purposes from a total of 55 clinically healthy horses of different breeds, age and sexes from 6 farms (farms 2 to 7) were used. A feeding anamnesis was recorded (Table 1).

### 5.2. Kinetics

These samples originated from a controlled study from 6 horses were leftovers from a project not subject to the present investigations [46]. In brief, 6 clinically healthy, warm-blooded mares (age: 6–13 years) with a mean body weight (**bw**) of 529 ± 38.7 kg were used. Blood samples were collected prior to offering 1 kg of meadow hay after an overnight fasting for 12 h (−60 min sampling time). From this offered hay amount, horses consumed 97 ± 46 g per 100 kg bw. Sixty min after offering the hay and immediately prior to concentrate feeding a further blood sample was taken (0 min sampling time). Thereafter, each horse received 16 g corn spindle meal and 171 g oats per 100 kg bw. Further blood samples were collected 30, 60, 90, 120, 180, 240 and 300 min after concentrate feeding. All blood samples were collected into serum tubes with clot activators and left at 4 °C for 30 min before being centrifuged at 2054 g for 10 min. Serum samples were stored at −20 °C until analysis.

Based on the DON and ZEN concentrations of the individual feedstuffs and the consumed amounts the ration contained 19 ± 2 µg ZEN and 512 ± 95 µg DON/kg feed corresponding to an exposure of 53 ± 4 ng ZEN/kg bw and 1420 ± 3 ng DON/kg bw, respectively.

### 5.3. Analyses

#### 5.3.1. Mycotoxin Residues

High-performance liquid chromatography with diode array detection (HPLC-DAD) and fluorescence detection (HPLC-FLD) were used to analyze DON and ZEN in feedstuffs, respectively, following the methods described by [47,48] with slight modifications as outlined by [49]. The limits of detection (LOD defined by a signal to noise ratio, S/N, of 3) were 15 and 1.2 μg/kg and the limits of quantification (LOQ, S/N = 10) amounted to 50 and 4.0 μg/kg for DON and ZEN, respectively. The corresponding mean recovery rates were approximately 93% and 92% for DON and ZEN, respectively.

Serum samples were incubated overnight with β-glucuronidase (Type H-2 from Helix pomatia, Sigma-Aldrich, Steinheim, Germany) to measure the sum of conjugated and unconjugated ZEN, DON and their metabolites. Afterwards, the samples were cleaned-up by solid phase extraction using Oasis HLB cartridges (Waters, Milford, MA, USA). ZEN, DON and their metabolites were analyzed by LC-MS/MS using a Pursuit XRs Ultra 2.8 C18 (100 × 2 mm, Agilent Technologies, Böblingen, Germany) for chromatographic separation [50].

The LODs ranged between 0.01 and 0.30 ng/mL. Specified LODs and LOQs (Figure 1F), as well as the mean rate of recovery for each analyte are published by [27].

#### 5.3.2. Trp and Kyn

Trp and Kyn concentrations were analyzed in serum samples as described by Hüther et al. [44]. Briefly, serum samples were mixed with ice cold ethanol for protein precipitation and fat extraction was performed by using hexane. After centrifugation (20,800× *g*), the supernatant was quantitatively transferred into a flask and evaporated in a nitrogen stream at 40 °C. The residue was dissolved in aqueous mobile phase A and after filtration (amcro filter, PVDF, 0.45 µm) 20 µL were injected into a HPLC system (Shimadzu, Kyoto, Japan). Separation of both metabolites was performed using a reversed phase C18-column (Inertsil ODS-2, 150 mm × 3.0 mm i.d., 5 μm particle size, Agilent; Boblingen, Germany) with a gradient elution. Mobile phase A consisted of 10 mM sodium 1-hexanesulfonate monohydrate, 0.5% (*v*/*v*) o-phosphoric acid and 0.5% (*v*/*v*) acetonitrile in ultrapure water; mobile phase B consisted of 100% acetonitrile. Detection wavelengths were 278 nm and 360 nm for Trp and Kyn, respectively. LOQs (S/N = 10) were 0.43 µmol L-1 for Kyn and 3.40 µmol L-1 for Trp. The Kyn/Trp ratio was calculated based on their molar concentrations in serum.

#### 5.3.3. Clinical-Chemical Traits

The analysis of the clinical chemical parameters in serum were carried out using an automated system (IndikoTM Plus, Thermo Fisher Scientific Oy Ratastie 2, 01620 Vantaa, Finland). For total protein, albumin, AST and GGT test kits from Thermo Fisher Scientific (article number 981827, 981767, 981771 and 981377, respectively, Thermo Fisher Scientific Oy Ratastie 2, 01620 Vantaa, Finland) were used while for GLDH a test kit from Labor+Technik (LT-GD0010, Labor+Technik Eberhard Lehmann GmbH, Berlin, Germany) was applied.

### 5.4. Calculations and Statistics

All analyte concentrations which were lower than the corresponding LOD were set to zero before statistical evaluation. Mycotoxin concentrations ranging between the LOD and LOQ were used for evaluation without further corrections. Beta-ZEL concentrations were additionally expressed as percentage of the sum of all detected ZEN metabolites as an indicator for the degree of metabolism of the parent compound ZEN.

Significant effects and trends were generally accepted at *p*-values lower than 0.05 and between 0.05 and 0.1, respectively, for all tests applied. All statistics were performed using the software package STATISTICA 12.0 [51].

#### 5.4.1. Screening

Mycotoxin residues and clinical-chemical traits in serum were not normally distributed and were consequently evaluated by non-parametric methods. First, box-whisker plots were created indicating the median, the span between the 25th and 75th percentile (boxes), and the range between minimum and maximum values as whiskers. Next, samples from the farms were evaluated whether they originated from the same distribution using the Kruskal–Wallis test by ranks followed by multiple comparisons.

Trp, Kyn and Kyn to Trp ratio were normally distributed and thus evaluated by analysis of variance (ANOVA) with farm as fixed factor followed by Tukey test for multiple comparisons in case of significant treatment effects.

The associations between all serum variables were further evaluated by PCA based on correlations. By reducing the dimensionality through creating new uncorrelated variables termed principal components (PC) [52] it becomes possible to absorb as much as possible variation from each individual original variable caused by treatment (farm). As PCs successively extract variation from the dataset the first two PCs are most informative for the whole dataset of the initial 13 variables. Original variables and cases (individual horses) were plotted between these first 2 PCs to visualize effects of variables and treatments (farms). Furthermore, newly constructed variables PC1 and PC2 were subjected to a multivariate analysis of variance (MANOVA) for detecting the effects of farms and of horse status (healthy, sick).

#### 5.4.2. Kinetics

Normally distributed parameters Trp, Kyn and Kyn to Trp ratio were analyzed through ANOVA with time relative to concentrate feeding as fixed factor for repeated measurements (same horses) and consideration of the values at −60 min as co-variates. In case of significant time effects least significant difference test was performed to identify significant differences between particular time points.

Mycotoxin residues were evaluated in a different way because of a large number of concentrations lower than the LOD. Here, only values greater than the LOD were evaluated across all horses and time points to identify any time effect through regressing time vs. mycotoxin residues. Next, the slopes of the simple linear regressions were tested for significant deviation from zero.

## Figures and Tables

**Table 1:** Assignment of horse patients with colic and clinically healthy horses to a farm number and information on feeding conditions.

Farm	Group	N	Feeding Conditions		
			Concentrate	Roughage	Pasture Access
1	Colic	102	No information		
2	Healthy	12	Oats, pellets, soybean meal	Grass silage	Yes
3	Healthy	10	Oats, muesli	Hay	Temporary
4	Healthy	10	Mix: 50% oats, 23% barley, 21% corn, 2.5% wheat bran, 2.5% mineral feed, 1% soybean oil	Grass silage	Yes
5	Healthy	7	Barley, oats	Hay	Yes
6	Healthy	8	Oats	Hay	Yes/no
7	Healthy	8	-	-	Yes

## Data Availability

Data sharing not applicable.
